# Development, Optimization, and In Vitro Evaluation of Novel Oral Long-Acting Resveratrol Nanocomposite In-Situ Gelling Film in the Treatment of Colorectal Cancer

**DOI:** 10.3390/gels7040276

**Published:** 2021-12-20

**Authors:** Shadab Md, Samaa Abdullah, Nabil A. Alhakamy, Waleed S. Alharbi, Javed Ahmad, Rasheed A. Shaik, Mohammad Javed Ansari, Ibrahim M. Ibrahim, Javed Ali

**Affiliations:** 1 Department of Pharmaceutics, Faculty of Pharmacy, King Abdulaziz University, Jeddah 21589, Saudi Arabia; nalhakamy@kau.edu.sa (N.A.A.); wsmalharbi@kau.edu.sa (W.S.A.); 2 Center of Excellence for Drug Research & Pharmaceutical Industries, King Abdulaziz University, Jeddah 21589, Saudi Arabia; 3 Mohamed Saeed Tamer Chair for Pharmaceutical Industries, King Abdulaziz University, Jeddah 21589, Saudi Arabia; 4 Department of Biological Sciences, Faculty of Science, King Abdulaziz University, Jeddah 21589, Saudi Arabia; 5 Department of Pharmaceutics, College of Pharmacy, Najran University, Najran 11001, Saudi Arabia; jaahmed@nu.edu.sa; 6 Department of Pharmacology & Toxicology, Faculty of Pharmacy, King Abdulaziz University, Jeddah 21589, Saudi Arabia; rashaikh1@kau.edu.sa; 7 Department of Pharmaceutics, College of Pharmacy, Prince Sattam Bin Abdulaziz University, Al-Kharj 11942, Saudi Arabia; javedpharma@gmail.com; 8 Department of Pharmacology, Faculty of Medicine, King Abdulaziz University, Jeddah 21589, Saudi Arabia; imibrahim1@kau.edu.sa; 9 Department of Pharmaceutics, School of Pharmaceutical Education and Research, Jamia Hamdard, New Delhi 110062, India; jali@jamiahamdard.ac.in

**Keywords:** resveratrol, soy protein, alginate film, nanocomposites, in-situ gel, oral sustained-release film, colorectal cancer

## Abstract

This study aimed to develop and evaluate sustained-release (SR) long-acting oral nanocomposites in-situ gelling films of resveratrol (Rv) to treat colorectal cancer. In these formulations, Rv-Soy protein (Rv-Sp) wet granules were prepared by the kneading method and then encapsulated in the sodium alginate (NA) dry films. The prepared nanocomposite in-situ gels films were characterized using dynamic light scattering, Fourier-transform infrared spectroscopy, X-ray diffraction, and scanning electron microscopy. The optimized formulations were further evaluated based on drug encapsulation efficiency, pH-drug release profile, swelling study, and storage time effects. The optimized formulation was tested for its anticancer activity against colorectal cancer cells using the cytotoxicity assessment, apoptosis testing, cell cycle analysis, gene expression analysis, and protein estimation by the reverse-transcriptase polymerase chain reaction and enzyme-linked immunosorbent assay methods, respectively. The optimum film showed encapsulation efficiency of 97.87% ± 0.51 and drug release of 14.45% ± 0.043 after 8 h. All physiochemical characterizations confirmed, reasoned, and supported the drug release experiment’s findings and the encapsulation assay. The Rv nanocomposite formulation showed concentration-dependent cytotoxicity enhanced apoptotic activity as compared to free Rv (*p* < 0.05). In addition, Rv nanocomposite formulation caused a significant increase in Bcl-2-associated protein X (*Bax*) and a decrease in expression of B-cell lymphoma 2, interleukin 1 beta, IL-6, and tumor necrosis factor-alpha (*Bcl2*, *IL-1β*, *IL-6*, and *TNF-α* respectively) compared to that of free Rv in HCT-116 cells. These results suggest that long-acting Rv nanocomposite gels could be a promising agent for colorectal cancer treatment.

## 1. Introduction

Resveratrol (3,5,4-trihydroxytrans-stilbene [Rv]) is a polyphenolic compound extracted from red grapes seeds that showed various pharmacological activities, such as anticancer and antioxidant and anti-inflammatory properties [1,2]. Rv has free radical scavenging activity to achieve its cancer-protective effects [1,2,3]. Regarding Rv’s effects on gene expression, Rv induces activation of caspases-3 and -9 and enhancement of B-cell lymphocyte-associated protein X (Bax) and tumor protein 53 (P53) expression to trigger apoptosis of cancerous cells [4,5]. Regarding the immuno-modulatory effects of Rv, triggering the secretion of interferon-gamma (IFN-ϒ) is a crucial activator of the immune system cytotoxic lymphocytes and T-helper and natural killer cells to capture and limit the growth of the cancerous cells [4,5,6,7]. Furthermore, Rv has an inhibitory role in the vascular endothelial growth factor (VEGF) activation and the release of reactive nitrogen species, which are secreted by the cancerous cells to enhance angiogenesis and create an inflammatory environment. Rv is considered an anti-inflammatory agent because of its negative effects on nuclear factor-kappa beta (NF-kB) and cyclo-oxygenases-1 and -2 (COX-1 and -2, respectively) as described in several studies [8,9,10]. All of these studies provide examples of the biological activities of Rv against cancer cells. Therefore, Rv is an excellent target to be incorporated into a pharmaceutical formulation for testing against colorectal cancer.

On the other hand, Rv presents several disadvantages, such as low bioavailability, high metabolism, and instability. High Rv metabolism, especially after oral ingestion, decreases its oral bioavailability and increases the need for repetitive administration, and is associated with Rv adverse effects and diminished patient adherence and compliance [1,2,3,8,10]. Additionally, Rv is prone to chemical and physical degradation during storage [4].

The soy protein (Sp) amount was optimized based on its capability of enhancing Rv stability [2], making it less subject to be metabolized [4,10] and enhancing immune stimulation for use in cancer management [11]. Additionally, Sp was used for its mucosal adhesive property to increase the Rv cellular uptake, absorption, and localization on the intestinal–colonic area after Sp-Rv wet granule release from the sodium alginate (NA) gel at pH values > 6.5 [12,13].

NA was used as a deposition film for its capability of encapsulating the drug by the conversion of the alginate films into alginic acid gels during contact with simulated gastric media (0.1 N HCl), as described by Bani–Jaber and Abdullah (2020) [14]. The potential interaction of NA with Rv was a possible retardant for Rv release from the Sp-Rv granules. Additionally, the Sp might strengthen the alginic acid gel activity when the Rv was freed from the wet granule Sp.

The novelty of the study is that no previous work was undertaken on the development of a sustained release oral long-acting resveratrol nanocomposite in-situ gelling film in the treatment of colorectal cancer. The novelty also includes a development of long-acting nanoparticles without the use of the organic solvents and a two-layer system comprising soy protein (1st layer) and sodium alginate (2nd layer). The specific sequence of the layers provides long-acting system targeting of the intestinal area as the site of absorption or action which is useful for colorectal cancer. The concept is new and merits further research, as it holds much promise for treating colorectal cancer that are producing significant rises in healthcare costs on a global scale.

The aim was to develop a long-acting sustained-release (SR) oral solid dosage form, which had the pH-selective release and mucoadhesive behavior at the intestinal–colonic area for the treatment of colorectal cancer. Additionally, the study aimed to enhance physiochemical storage stability for Rv. The formulations were planned to use Sp-Rv wet granulation and NA encapsulation. The combination amounts of the constituents and preparation method were optimized to develop long-acting SR oral formulations. These formulations were intended to encapsulate the drug as a nanocomposite in an in-situ gelling film. The film was designed to be given after water reconstitution or filled in hard gelatin capsules. Therefore, the objective of the present study was to develop and optimize SR long-acting oral nanocomposites in in-situ gelling film of Rv for colorectal cancer treatment. The prepared nanocomposite in-situ gels films were characterized using dynamic light scattering, Fourier-transform infrared spectroscopy, X-ray diffraction, and scanning electron microscopy (FTIR, XRD, and SEM, respectively). The optimized formulations were evaluated based on drug encapsulation efficiency, pH-drug release profiles, swelling studies, and storage time effects. The optimized formulation was tested for its anticancer activity against colorectal cancer cells using the cytotoxicity assessment, apoptosis testing, cell cycle analysis, gene expression analysis, and protein estimation by the real-time polymerase chain reaction and enzyme-linked immunosorbent assay (RT-PCR and ELISA, respectively) methods, respectively.

## 2. Results and Discussion

### 2.1. Formulation Optimization of Nanocomposite Film

The nanocomposite film was prepared in two different stages, consisting of Sp and Rv kneading (using two ratios of 3 Sp:5 Rv, and 6 Sp:5 Rv) and encapsulation of the Sp-Rv granules in the NA gels (using 0.43 and 0.52 g/g of the product weight, respectively) via a slow dripping and mixing method. The most optimum film was the NA-Sp-Rv-1200 containing 0.52 g NA and 0.26 g Sp/g of the film.

### 2.2. Drug Release Analysis and Drug Encapsulation Assay

Using the pH-profile drug release, the amounts of NA and Sp and film preparation method were optimized to achieve a minimum release in the stomach-simulated media and sustained-release (SR) behavior in the intestinal and colonic media. The SR system of the pH-selective release was aimed to decrease the metabolism associated with the free drug and to decrease the number of daily doses to enhance the efficiency associated with the Rv use as anti-inflammatory or anticancer treatments [15,16,17,18,19,20].

**Figure 1:**
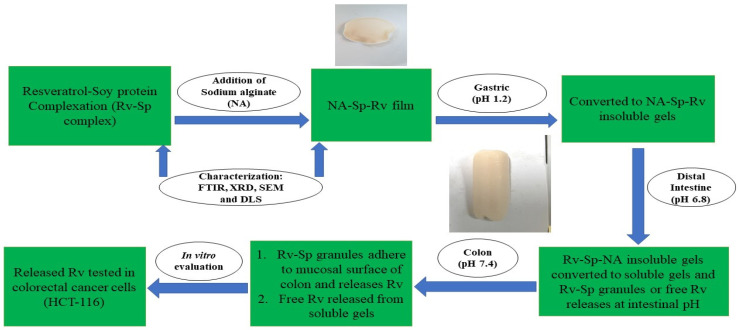
Illustration of long-acting resveratrol nanocomposite in-situ gelling film mechanistic release.

The optimized film composed of resveratrol-soy protein complex encapsulated in sodium alginate was developed for oral administration. Afterwards the film is converted into insoluble gels in contact with simulated gastric media (pH 1.2). The insoluble gels are formed due to the conversion of sodium alginate into insoluble alginic acid at gastric pH. The insoluble gels strength was also enhanced due to presence of soy protein sodium alginate interaction. After that, the insoluble NA gels get converted into soluble gels due to change in pH, to release the Sp-Rv granules or the free Rv at the intestinal and colonic simulated media [13,14,15]. The Sp-Rv nanoparticles adhere to the colonic mucosal surface for better cellular uptake and cells internalization. This adherence was due to soy protein mucoadhesive nature and neutral zeta potential values of the nanoparticles [11,13]. The pH selective release of Rv nanoparticles from the prepared nanocomposite film would improve the Rv local action and sensitivity on the colon cancer cells, time-dependent efficacy, and decrease the Rv metabolism inside the gastrointestinal tract. Furthermore, Sp and NA boosts the humoral and cell mediated immunity of the body to enhance the cancer immunosurveillance [9,11]. The illustration of mechanism of long-acting resveratrol nanocomposite in-situ gelling film are explained in Figure 1.

For the most optimized formulation, NA-Sp-Rv-1200, the drug encapsulation efficiency was 97.87 ± 5.1%. Therefore, the drug encapsulation efficiency was used to calculate the drug’s percentage (%) release in each formulation in Table 1.

**Figure 2:**
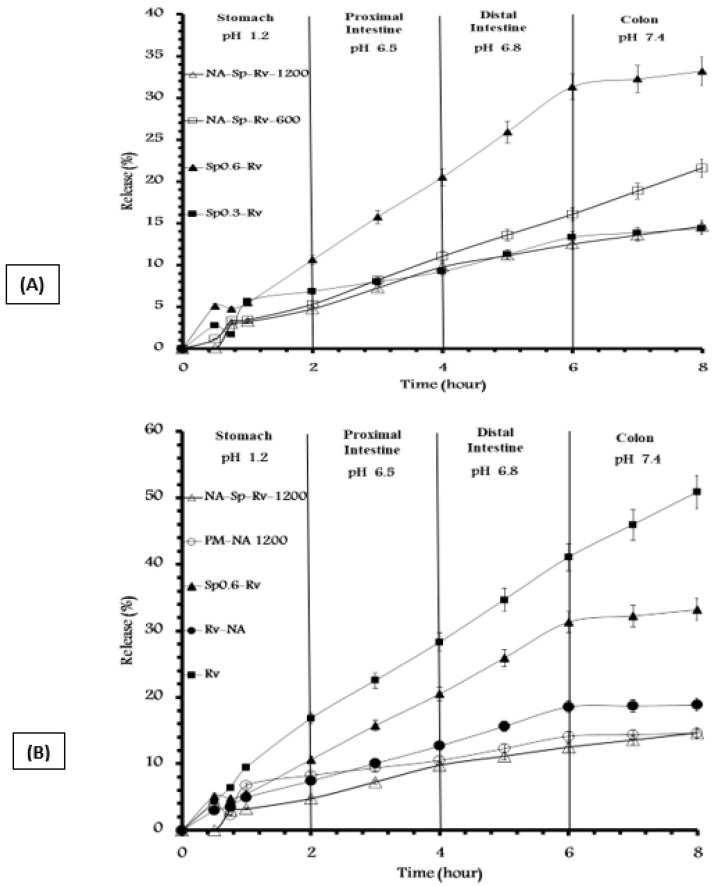
(**A**,**B**) pH profile release of different formulations containing 0.5 g of Rv.

The different formulations were compared with respect to the % gastric release, % intestinal-colonic release, and overall release as listed in Figure 2A,B. The resulting amounts of Sp were 0.26 and 0.21 g/g. The resulting amounts of Na were 0.52 and 0.43 g/g. The optimum NA and Sp amounts for achieving the intended SR profile, best drug encapsulation, and nanocomposite film formation were 0.52 g and 0.26 g/g of NA and Sp, respectively.

The difference between Sp0.3-Rv and Sp0.6-Rv is shown in Figure 2A. Sp 0.6-Rv caused a release of approximately double the gastric and intestinal–colonic releases of the Sp0.3-Rv, which might be an advantage when the Sp-Rv particles released from the NA matrix have aqueous release and absorption after the contact of cells in the presence of Sp as a mucoadhesive polymer. Furthermore, Rv release was higher than the Sp0.6-Rv release as suggested in Figure 2B, which was a good indication of the drug-Sp adsorption and revealed the hydrophilic functionalities of Rv.

Furthermore, the difference between NA-Sp-Rv-1200 and -600 was shown in Figure 2A. The NA-Sp-Rv-600 gastric release was the same as the NA-Sp-Rv-1200 (*p* > 0.05). On the other hand, the intestinal–colonic release of NA-Sp-Rv-600 was 1.6-fold that of the NA-Sp-Rv-1200 release, which indicated a better sustained-release profile in NA-Sp-Rv-1200; thus, it was considered the optimum formulation.

To study the effect and role of each component, the other formulations in Table 1 were examined for their pH-release profiles. The Sp effect was essential when comparing the release of Rv with Sp0.6-Rv. The Sp0.6-Rv gastric and intestinal–colonic release was less than the Rv gastric and intestinal–colonic release by 6.21 ± 0.041% and 11.48 ± 0.048%, respectively. This finding might be due to the Sp-hydrophobic amino acids surrounding the Rv, which hindered the drug release [4,12,20].

Additionally, the effect of Sp was noticeable when comparing the Rv-NA with the NA-Sp-Rv-1200. The NA-Sp-Rv-1200 gastric and intestinal–colonic release was less than the Rv-NA gastric and intestinal–colonic release by 2.66 ± 0.031% and 1.58 ± 0.048%, respectively. The same Sp hydrophobic interference might be the reason for this difference. The capability of Sp to strengthen the NA gel formation while in contact with the simulated gastric media is supported by FTIR results [12,13].

To study NA film effects, a comparison between NA-Sp-Rv-1200 films and Sp0.6-Rv granules or Rv was done. The NA-Sp-Rv-1200 gastric and intestinal–colonic release was less than the Sp0.6-Rv gastric and intestinal–colonic release by 5.85 ± 0.042% and 12.72 ± 0.041%, respectively. Additionally, the NA-Sp-Rv-1200 gastric and intestinal–colonic release was less than the Rv gastric and intestinal–colonic release by 12.06 ± 0.012% and 24.2 ± 0.015%, respectively. The minute amount of gastric release of the film and slow rate of the intestinal–colonic release resulted from the formation of the alginic acid gel in contact with 0.1 N HCl and the slow dissolution at pH values > 4.5, which released the Sp-Rv granules for Sp cellular adherence, uptake, or absorption [12,14,15,19]. The alginic acid gel formation with Sp gel strength enhancement, Sp hydrophobicity hindrance, faster dissolution of Sp0.6-Rv compared to Sp0.3-Rv, and Rv interaction with NA to capture the free Rv were the main reasons for the optimum SR capability of the NA-Sp-Rv-1200 film.

The effect of the unique formulation steps in NA-Sp-Rv-1200 was illustrated compared to the physical mixture of the same constituents (PM-NA 1200). The NA-Sp-Rv-1200 release was less than the gastric release of PM-NA 1200 by 3.49 ± 0.046% and more than the intestinal–colonic release of PM-NA 1200 by 3.51 ± 0.034%. The time needed for the NA to encapsulate Rv and Sp after wetting with 0.1 N HCl possibly caused an increase in the gastric release. On the other hand, the difference in the Sp and Rv physical mixture gel arrangements, which happened spontaneously depending on the hydrophilic/lipophilic balance, might have interfered with the Sp hydrophobic amino acids with the Rv inside the NA hydrogel in contact with the intestinal–colonic media. This process possibly triggered an intestinal-colonic release of PM-NA 1200 that was lower than the formulated film [12,13].

The rest of the Rv in the NA-Sp-Rv-1200 (85.55 ± 1.43%) would be released in the colon after 22 to 71 h of residency in the colon, which would depend on food ingestion and disease status [21,22]. The assumption was built on the [21,22] references related to the gastrointestinal residency time. Moreover, the rest of the optimum film release (Figure 2A) could be mathematically calculated because the release follows Korsmeyer–Peppas kinetics fitting equation for 0–2 h, and the Zero-order equation for 2–8 h (Table 2). As a result, the 100% release could be achieved at 71 h using the zero-order equation at 2 to 8 h, as NA-Sp-Rv-1200 film’s release curve is representing a positive linear pattern.

Mt/M∞ is a fraction of the drug released at the time (*t*), K is the release rate constant, and n is the release exponent. The n value is used to characterize different releases for cylindrical-shaped matrices. To determine the exponent of n, the portion of the release curve, where Mt/M∞ < 0.6, should only be used as log cumulative percentage drug release versus log time. The release mechanism is a function of the diffusion exponent n. For a thin-film geometry delivery system: *n* = 0.5 suggests a Fickian diffusion; 0.5 < *n* < 1.0 supports an anomalous non Fickian transport (both diffusional and relaxation transport); for *n* = 1.0, means zero-order release represented by a case-II, relaxational transport time-independent. Parameters of the fittings were reported in Table 3, and it is suggested during the first 2 h that the release was highly diffusion limited (Fickian diffusion) [23].

However, x is the time, y is drug dissolved percentage at time *t*, and a is the rate constant (h − 1) taken as the slope of the linear fitting with intercept (b) [23].

### 2.3. Storage Time Effect on the Nanocomposites of In-Situ Gelling Film

According to Table 3, the NA-Sp-Rv film was stable for up to eight weeks during storage, during which the average similarity factor (*F*2) compared to the freshly prepared film was 95.45 ± 0.31%. This finding might result from the NA and Sp capturing the Rv in a dry film form. In conclusion, this optimum formula might provide an excellent alternative for solving the Rv physical and chemical instability during storage [1]. The *F*2 value was calculated using the equation:

$$F2=50*log\left\{{\left(1+\frac{1}{P}*{\left(Rt-Tt\right)}^{2}\right)}^{-0.5}*100\right\}$$

In which *F*2 is the similarity factor, and *P* is the number of release samples. *Rt* and *Tt* are dissolution percentages of the compared products. The compared products were dissimilar for drug release performance when the *F*2 value was <50 [23].

### 2.4. Swelling Study of Nanocomposites of In-Situ Gelling Film-Rv Nanoparticle

**Figure 3:**
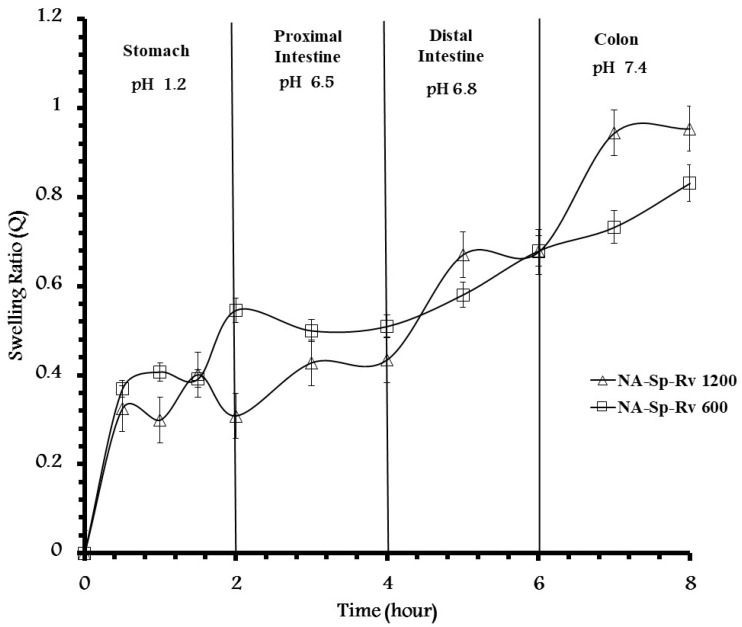
pH-effect on swelling ratio of NA-Sp-Rv-1200.

The swelling of the optimal in-situ gelling formula (Figure 3) showed increments in the swelling ratio with the increase in the pH from 1.2 up to 7.4. At the end of treatment, the swelling ratios for NA-Sp-Rv using 0.52 g/g NA and 0.26 g/g Sp were 0.31 ± 0.03, 0.44 ± 0.04, 0.68 ± 0.01, and 0.95 ± 0.03 at the pH values of 1.2, 6.5, 6.8, and 7.4, respectively. The increase in the swelling ratio confirmed the increase in drug release over the higher values of the pH profiles. As a result, the hydrodynamic gel structure of the Sp-NA in-situ gel was essential for developing the selective pH-SR system. As shown in Figure 3, fluctuations in the swelling ratio in the simulated gastric phase during the first 2 h might were caused by the equilibrium between the NA and alginic acid gel formation. It was confirmed by the kinetics fitting of Fickian diffusion [12,24]. These specific amounts of 0.52 g/g NA and 0.26 g/g Sp in the optimum formula were characterized as having a lower swelling ratio at pHs of 1.2 and 6.5 and higher swelling rates of change with more sensitivity to the pH change at pH 6.8 (area of Rv absorption) and 7.4 than the formula containing 0.43 g/g of NA and 0.21 g/g of Sp. The formula with 0.43 g/g of NA and 0.21 g/g of Sp (NA-Sp-Rv-600) showed a higher swelling ratio at pH 1.2 and 6.5 than the NA-Sp-Rv-1200 formula. Additionally, NA-Sp-Rv-1200 revealed a similar rate of swelling ratio change at pH 6.8 and 7.4 as found with pH values of 1.2 and 6.5 in the NA-Sp-Rv-600 [12,13,24]. However, the NA-Sp-Rv-600 showed more fluctuated swelling behavior than the NA-Sp-Rv-1200 swelling ratio, especially at the first 2 h. However, the NA-Sp-Rv-1200 film was considered optimum due to the stable swelling after 2 h in comparison to the NA-Sp-Rv-600 film [12,24].

### 2.5. Nanocomposites of In-Situ Gelling Films-Rv Nanoparticle and Sp-Rv Granules Characterizations

#### 2.5.1. Particle Size Distribution and Zeta-Potential Measurements

The average particle size of the Rv in the film was 392.8 ± 5.43 nm, which was considered a nano-drug composite film containing particles in the range of 1 to 1000 nm [25]. The particle size populations were found to be 392.8 ± 5.43 nm. These results were associated only with this ratio of combined constituents (2:1:0.9 of NA:Sp:Rv) and the preparation method resulting from the formula optimization. When the NA amount was increased, the shearing force in the mixing increased, and particle size reduction occurred [4,12,14,15]. The nanoparticle encapsulation of the drug and Sp inside the NA film would have resulted in the SR behavior results [13]. A zeta-potential value of −0.235 ± 0.012 mV might result from the nanoparticles’ NA coating (carboxylate negative charge), which was neutralized in the presence of Sp and Rv [12,13,15,26]. As a result, the electrical conductivity was 4.79 ± 0.12 mS/cm. The PDI was 0.488 indicating homogenous nanoparticles character [13]. Additionally, the nanoparticle aggregation happened when the Rv and Sp nanoparticles were released from the film because these zeta potentials and PDI values were related to the nanoparticles after release. On the other hand, these zeta values supported the concept of the released particle adhesiveness to the intestinal and colonic mucosal cells [13,26].

#### 2.5.2. Chemical and Physical Interactions Elucidation

**Figure 4:**
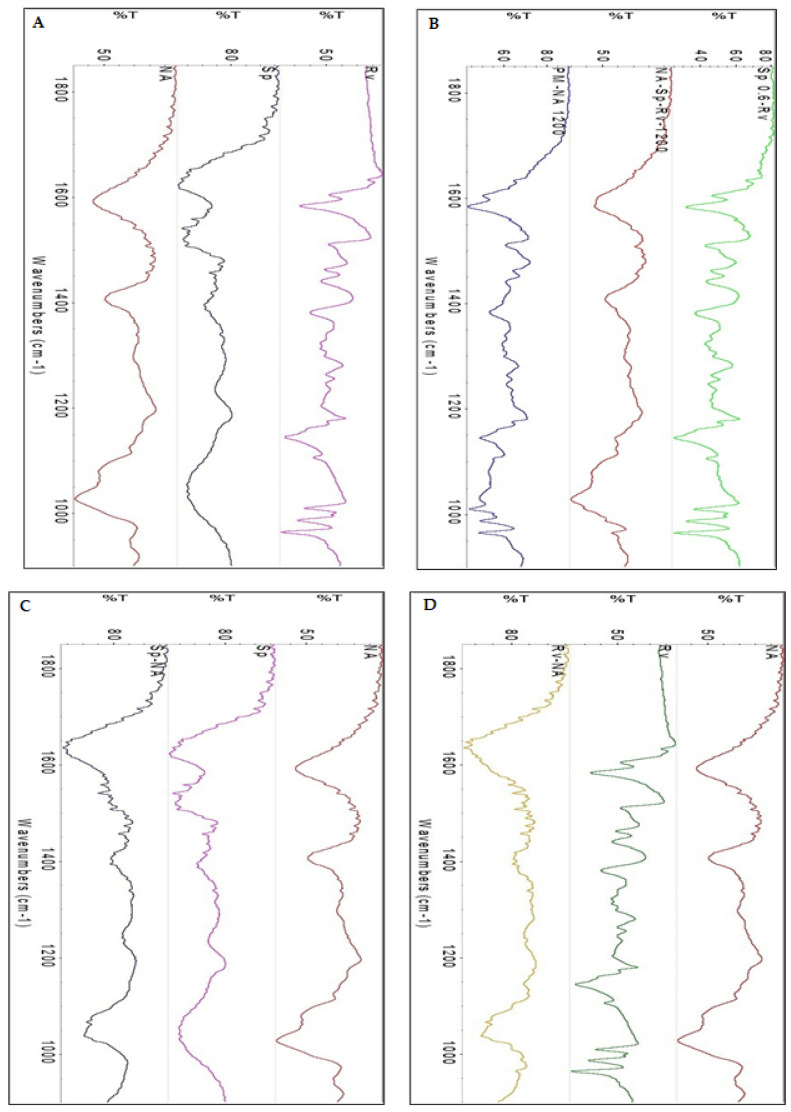
FT-IR spectra of Rv, Sp and NA (**A**), Sp0.6-Rv, NA-Sp-Rv-1200 and PM-NA 1200 (**B**), Sp-NA (**C**), and Rv-NA (**D**).

According to Figure 4A–D, FTIR spectra were obtained for Rv (Figure 4A), Sp (Figure 4A), NA (Figure 4A), Sp0.6-Rv (Figure 4B), PM-NA 1200 (Figure 4B), NA-Sp-Rv (Figure 4B), Sp-NA (Figure 4C), and Rv-NA (Figure 4D). The spectra were analyzed for the potential interactions between the constituents, which would have affected drug release and the other physical characteristics of the optimum nanocomposite film (NA-Sp-Rv). Regarding the Rv spectrum (Figure 4A), the carbon-carbon aromatic double-bond and carbon-carbon olefinic vibrations occurred at 1606 and 1587 cm^−1^, respectively. The benzene ring vibrations were revealed at 1513 and 1463 cm^−1^. The peak of 1384 cm^−1^ was attributed to a carbon-oxygen vibration. The carbon = carbon–hydrogen peak and demonstration of the “trans” form of resveratrol occurred peaks at 988 and 966 cm^−1^, respectively [15]. The spectrum of Sp (Figure 4A) with an amide I peak of the carbon-nitrogen bond was 1626.35 cm^−1^. The amide II band of the 2/3 nitrogen-hydrogen stretch and 1/3 carbon-nitrogen bond was found at approximately 1521.08 cm^−1^. The amide III band of the carbon-nitrogen and nitrogen–hydrogen bends occurred at approximately 1400 cm^−1^ [13]. The NA spectrum (Figure 4A) revealed essential peaks of hydroxyl, ether, and carboxylic functional groups. The 1595 cm^−1^ peak was related to asymmetric stretching vibrations of the carboxylate salt ion. This and the later peaks were essential and used to characterize alginate structure from its derivatives and ingredients. The carbon-oxygen bond peak of the pyranose ring, the carbon–carbon-hydrogen bond, and carbon–oxygen-hydrogen bond deformations occurred at 1400 and 1010 cm^−1^ [5,12,13]. The spectrum for the physical mixture (Figure 4B) of NA, Sp, and Rv (PM-NA 1200) revealed a blend of all constituent peaks. The spectrum of Sp0.6-Rv wet granules (Figure 4B) showed the Rv peaks with Sp peak disappearance. This disappearance might have resulted from the Sp surfaces’ Rv deposition or the Sp dispersion and particle size reduction of Sp between the Rv larger particle [26]. The in-situ gelling films of the NA-Sp-Rv spectrum (Figure 4B) represented the NA same essential peaks with 966 cm^−1^-Rv peak. This gelling would have led to the conclusion of the NA encapsulation or the particle size reduction of Rv and Sp inside the gel, which could be confirmed by the SEM result as described in the next section [26].

For elucidation of the expected molecular interaction and arrangements between NA and Sp in the in-situ gelling film, the Sp-NA film spectrum (Figure 4C) showed the disappearance of NA-1595 cm^−1^ peak of carboxylate functionality, the absence of NA peaks at wavelengths ranging from 1390 to 1190 cm^−1^, and the presence of the broad NA-1010 cm^−1^ peak of the pyranose ring functionalities with lower intensity. On the other hand, the Sp peaks of 1626 cm^−1^ and Sp peaks in the range of 1390 to 1190 cm^−1^ were apparent in the Sp-NA spectrum. The 1520 cm^−1^ Sp peak was absent in the Sp-NA spectrum. The disappearance of the NA-carboxylate functionality peak and the Sp-amide-II peak indicated potential interaction (such as a hydrogen bond) between these functionalities inside the film, which could be attributed to Sp strengthening the binding capacity toward NA [12,13]. The rest of the peaks of certain functionalities might were located on the surface of the film. However, the absent ones might be folded inside the film structure [26].

To elucidate the interactions between Rv and NA after the release from the Sp0.6-Rv wet granules, the Rv-NA film spectrum (Figure 4D) revealed a new broad peak at 1620 cm^−1^, which might be the result of merging Rv and NA essential peak at this range. The Rv-NA film showed the absence of Rv peaks with NA peaks in the range of 1550 to 1190 cm^−1^ with significantly diminished intensities. This finding could be attributed to the interactions between the Rv structure and NA-hydroxyl and carboxylate functionalities, incorporation of Rv inside the NA film, and the particle size reduction of Rv inside the NA gel [5,15].

#### 2.5.3. X-ray Diffraction of the Wet Granules and In-Situ Gelling Films

**Figure 5:**
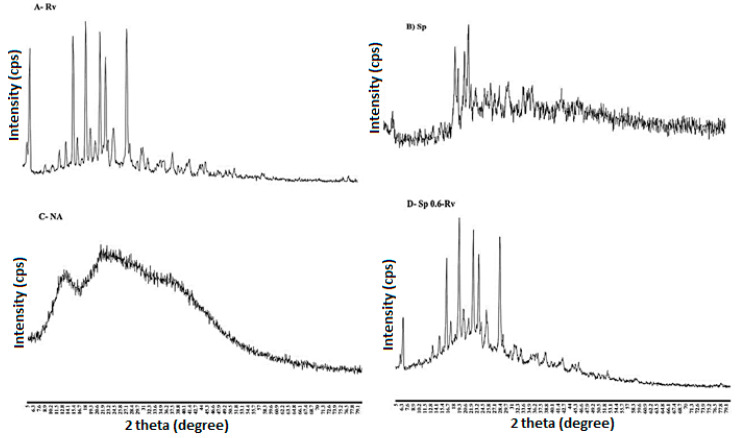

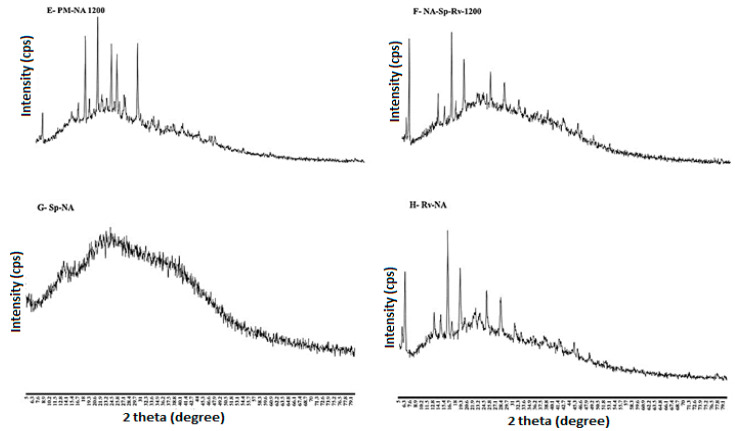
PXRD diffractograms of Rv (**A**), Sp (**B**), NA (**C**), Sp0.6-Rv (**D**), PM-NA 1200 (**E**), NA-Sp-Rv-1200 (**F**), Sp-NA (**G**), and Rv-NA (**H**).

Powder XRD of Rv, Sp, NA, Sp0.6-Rv, PM-NA 1200, NA-Sp-Rv-1200, Sp-NA, and Rv-NA are shown in Figure 5A–H. In Figure 5A, the Rv powder showed well-defined peaks in the studied range, which might indicate the crystalline nature of the Rv powder. Regarding Sp (Figure 5B) and NA (Figure 5C), these two compounds did not show specific diffraction peaks, which could be attributed to their amorphous natures [12,13]. On the other hand, the wet granules (Figure 5D) of Sp0.6-Rv showed some of the well-defined peaks of Rv with the dominant amorphous nature of Sp. The physical mixture of the previous raw materials (PM-NA 1200) showed some of the Rv characteristic diffraction peaks with noisy NA and Sp peaks, as seen in Figure 5E. The powder XRD of the NA-Sp-Rv-1200 film (Figure 5F) confirmed the amorphous nature of the particles. To understand the interaction in the NA-Sp-Rv-1200 film, the Sp-NA film (Figure 5G) had more apparent amorphous/irregular shape properties than the Rv-NA film (Figure 5H). As a result, the Sp and NA natures and interactions were shown in Sp-NA, which it might have contributed to the NA-Sp-Rv-1200 film amorphic character with some Rv well-defined peaks [12,13,15].

#### 2.5.4. Dispersion, Morphology, and Distribution of the Wet Granules and In-Situ Gelling Films

**Figure 6:**
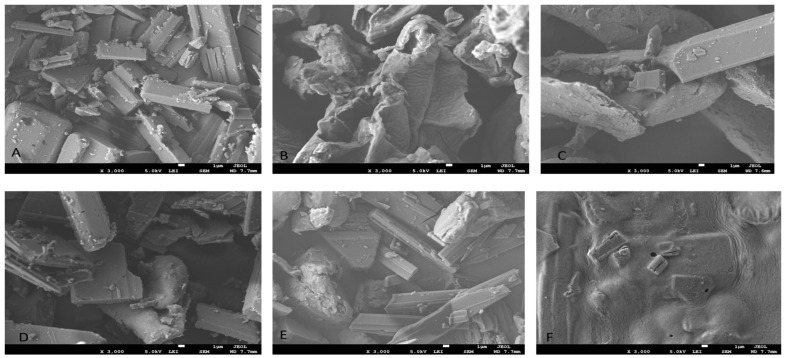
SEM morphological distributions of Rv (**A**), Sp (**B**), NA (**C**), Sp0.6-Rv (**D**), PM-NA 1200 (**E**), and NA-Sp-Rv-1200 (**F**).

Using SEM, the size distribution, shape, and dispersion of Rv, Sp, NA, Sp0.6-Rv, PM-NA 1200, and NA-Sp-Rv-1200 films can be observed in Figure 6A–F. Rv rectangular crystals were observed (Figure 6A). On the other hand, the Sp powder appeared to have a non-uniform shape (Figure 6B). The NA powder was found to possess a non-uniform shape, which is shown in Figure 6C. In Figure 6D, the wet granules of Sp0.6-Rv comprised the Sp powder and the Rv rectangular particles. The physical mixture picture (Figure 6E) showed the three constituents with their unique shapes. The NA-Sp-Rv confirmed the film encapsulation (Figure 6F).

### 2.6. In Vitro Cell-Line Evaluation

#### 2.6.1. Cell-Viability Analysis Using MTT Assay

**Figure 7:**
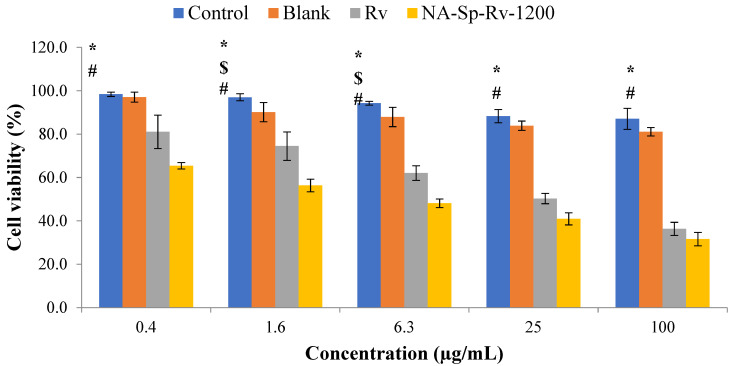
Cell-viability analysis of Rv, NA-Sp-Rv-1200, Blank, and control after 24 h (statistical inferences (*p* < 0.05) between NA-Sp-Rv-1200 film compared with control (#), NA-Sp-Rv-1200 film compared with blank ($), and NA-Sp-Rv-1200 film compared with Rv (*)).

As shown in Figure 7, the Rv and its nanocomposite film presented cell cytotoxicity as being concentration-dependent. This finding confirmed Rv’s anticancer activity [7,8]. On the other hand, the NA-Sp-Rv-1200 film showed a significant increase in cytotoxicity compared to control and free Rv at different concentrations. The improved anticancer properties of the NA-Sp-Rv-1200 film were possibly due to better cellular uptake of nanoparticles, and NA with Sp could contribute to the superior anticancer properties [9,13,19]. Additionally, the calculated IC_50_ of the Rv was 55.44 ± 1.3 µg/mL, which was more than the film’s IC_50_ of 20.80 ± 1.5 µg/mL. However, the differences between the blank and control cell viabilities were added to understand the NA and Sp contribution in the anticancer action at those concentrations. The differences between the control and the blank over concentrations of 0.4, 1.6, 6.3, 25, and 100 µg/mL were 1.05% ± 1.1%, 2.94% ± 3.6%, 1.5% ± 2.1%, 5.41% ± 1.8%, and 6.59% ± 1.5%, respectively. These differences contributed to the other polymers that could be correlated with better anticancer actions compared to that of the free drug. Still, the major contribution was suggested to originate from the Rv nanoparticles with better sensitivity and efficacy [26].

#### 2.6.2. Apoptosis Analysis & Cell-Cycle Analysis Using Annexin V-Propidium Iodide Assay

**Figure 8:**
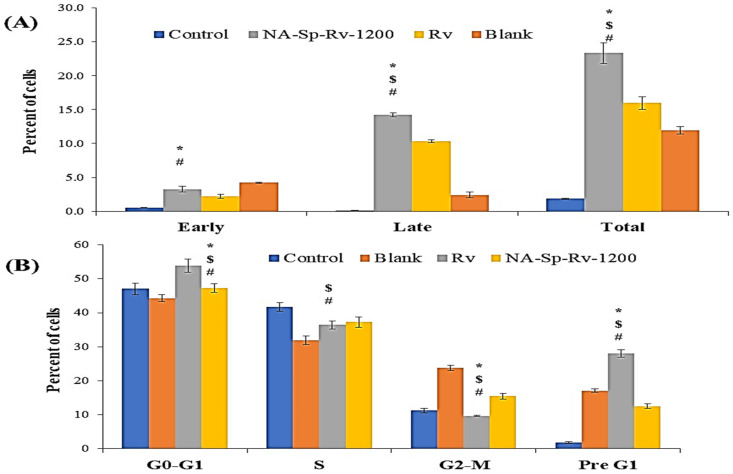
Apoptosis (**A**) and cell-cycle analysis (**B**) of Rv, NA-Sp-Rv-1200, Blank, and control after 24 h (Statistical inferences (*p* < 0.05) between NA-Sp-Rv-1200 film compared with control (#), NA-Sp-Rv-1200 film compared with blank ($), and NA-Sp-Rv-1200 film compared with Rv (*)).

As presented in Figure 8A, Rv-free drug-induced apoptosis was more than the percentage apoptosis of the control group by 14.12 ± 0.31%, a finding that supported the previously published data [7,9]. On the other hand, the NA-Sp-Rv-1200 film presented a significant increase in apoptosis compared to that of the control group (21.48 ± 0.23%), which was consistent with the MTT results. Nanocomposite films showed significant increases in apoptosis compared to Rv due to the contribution of Rv nanoparticles and the polymeric matrix in enhancing free drug efficacy and sensitivity. Additionally, the late apoptosis event was predominant in both Rv and NA-Sp-Rv-1200 film, suggesting that Rv and its nanoparticles were the triggers for apoptosis induction. However, the blank-induced apoptosis was more than the percentage apoptosis of the control group by 10.06 ± 0.44, which confirmed the matrix contribution in more intense apoptosis of the film, especially early apoptosis. Furthermore, NA was evaluated for its anticancer activity, and Sp, with its cell-adhesion and hydrophobic nature, could have triggered hypoxia of the cancer cells and disturbances in the cell membrane [12,13,20].

Two types of apoptosis were found: (1) cell-death-induced apoptosis and (2) cell cycle delay-induced death. Furthermore, the cell residency in the pre-G1 and -G2-M phases indicated the cell cycle’s shift from replication to aging and death [25]. As shown in Figure 8B, the percentage of G2-M and pre-G1 after treatment with Rv, NA-Sp-Rv-1200, Blank, and control were 37.66 ± 1.11%, 28.01 ± 0.67%, 40.89 ± 0.14%, and 13.07 ± 0.56%, respectively. Compared to that of the control, the highest G2-M and pre-G1 values were found in the blank group, followed by the Rv and then by the NA-Sp-Rv-1200 groups. Cell cycle delay-induced apoptosis in the blank group was suggested to originate from the NA polymer [9].

On the other hand, the Sp participated in inducing the early apoptosis as suggested in the apoptosis analysis. However, the Rv group had a cell cycle delay effect that was stronger than the NA-Sp-Rv-1200 group by approximately 3%, which correlated with the slow-release system in the NA-Sp-Rv-1200. Additionally, the free drug available in NA-Sp-Rv-1200 was lower than in the Rv group, but the cell-cycle results were still approximately the same for both groups. Thus, the nanoparticles and the polymeric matrix in NA-Sp-Rv-1200 might have compensated for the high Rv amount in the free drug group.

#### 2.6.3. Gene Expression Analysis Using RT-PCR

**Figure 9:**
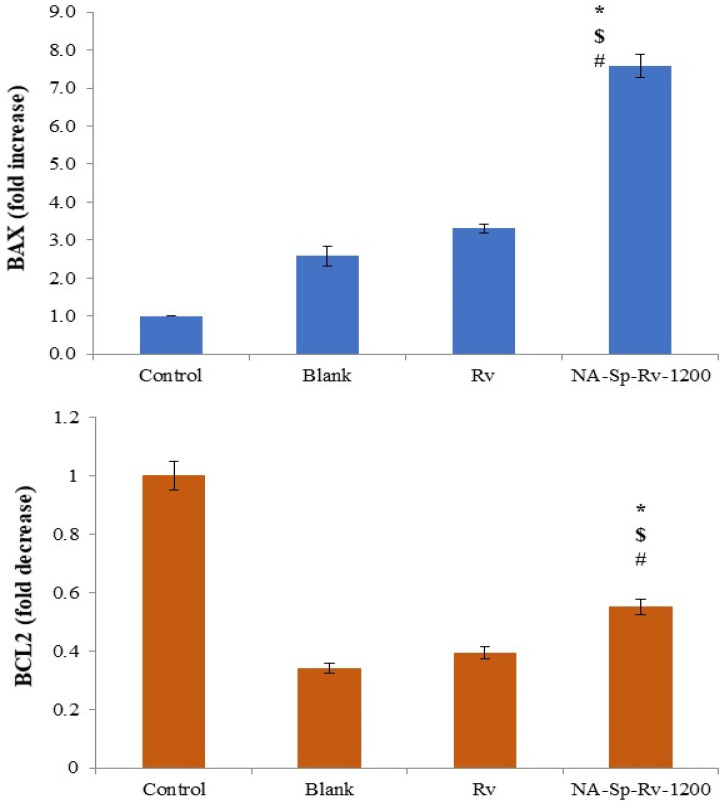
Gene expression analysis of *BAX*, and *BCL-2* of Rv, NA-Sp-Rv-1200, Blank, and control after 24 h (Statistical inferences (*p* < 0.05) between NA-Sp-Rv-1200 film compared with control (#), NA-Sp-Rv-1200 film com-pared with blank ($), and NA-Sp-Rv-1200 film compared with Rv (*)).

Regarding the *BAX* as a proapoptotic gene, the expression folds increase for NA-Sp-Rv-1200 was nearly double, triple and seven folds of the Rv, blank, and control folds increase, respectively (Figure 9). On the other hand, the folds decrease for NA-Sp-Rv-1200 of *BCL2* expression (Anti-apoptotic gene) were nearly 1.45, and 1.6 folds more than the Rv and blank, respectively. These gene expression results were suggesting enhanced apoptosis in NA-Sp-Rv-1200 on the molecular levels, and it came supportive for the previous evaluations [8,9,27,28].

**Figure 10:**
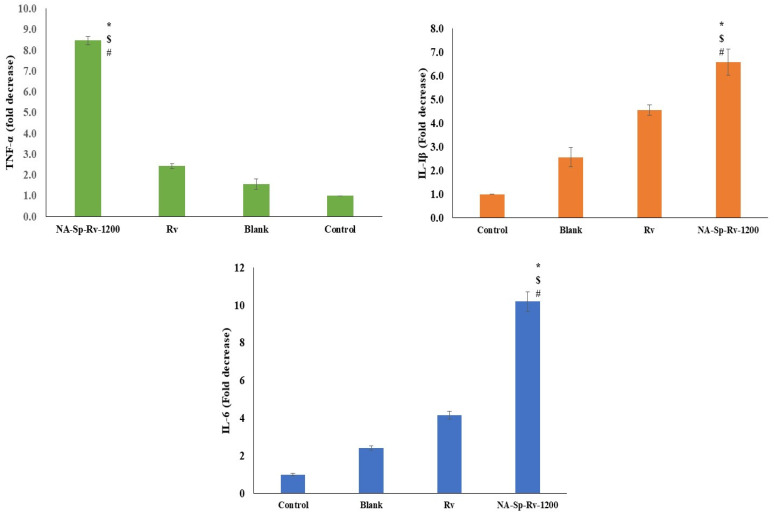
Gene expression analysis of *TNF-α*, *IL-1β*, and *IL-6* of Rv, NA-Sp-Rv-1200, Blank, and control after 24 h (Statistical inferences (*p* < 0.05) between NA-Sp-Rv-1200 film compared with control (#), NA-Sp-Rv-1200 film com-pared with blank ($), and NA-Sp-Rv-1200 film compared with Rv (*)).

The *TNF-α* gene expression for the nanocomposite film was less than the Rv, blank, and control by nearly four, six, and eight folds, respectively. Additionally, the *IL-1β* gene expression for the nanocomposite film was less than the Rv, blank, and control by nearly two-, three-, and seven-fold, respectively. Finally, the *IL-6* gene expression for the nanocomposite film was less than the Rv, blank, and control by nearly three, five, and ten folds, respectively. Afterall, *TNF-α*, *IL-1β*, and *IL-6* genes were coding for the proinflammatory cytokines as TNF-α, IL-1β, and IL-6 (Figure 10) [8]. The Rv was approved for its anti-inflammatory and proapoptotic actions on the cells, which was the best in the NA-Sp-Rv-1200 [9]. Thus, the NA-Sp-Rv-1200 film results could be attributed to the better sensitivity and efficacy of the nanoparticles [13].

#### 2.6.4. Protein Concentration Assessment Using ELISA

**Figure 11:**
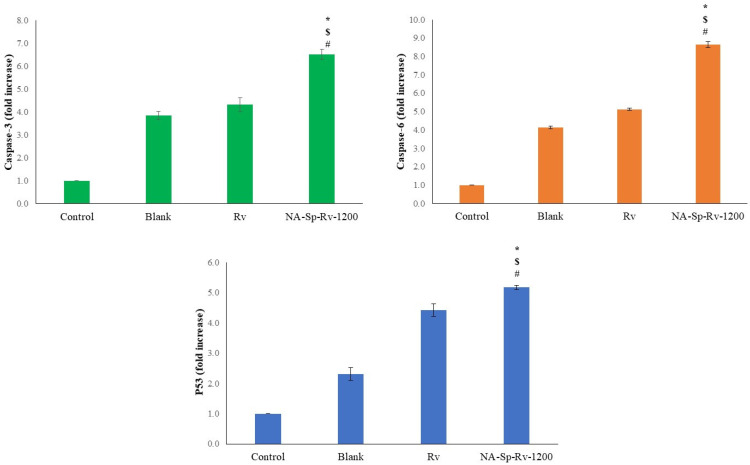
Protein concentration analysis of Caspase-3, Caspase-6, and P53 of Rv, NA-Sp-Rv-1200, Blank, and control after 24 h (Statistical inferences (*p* < 0.05) between NA-Sp-Rv-1200 film compared with control (#), NA-Sp-Rv-1200 film compared with blank ($), and NA-Sp-Rv-1200 film compared with Rv (*)).

Regarding Caspase-3 expression as a proteolytic enzyme, the folds increase for the NA-Sp-Rv-1200 was nearly 1.67, 1.54 and 6.51 folds of the Rv, blank, and control levels, respectively. On the other hand, Caspase-9 expression as a proteolytic enzyme, the folds increase for the NA-Sp-Rv-1200 was nearly 1.68, 2.08 and 8.63 folds of the Rv, blank, and control levels, respectively. Additionally, the P53 protein expression for the NA-Sp-Rv-1200 was more than those for the Rv, blank, and controls by 1.17, 2.38, and 5.18 folds, respectively (Figure 11). However, the optimum film drug release was less than the Rv group, but the anticancer and apoptotic molecular indicators were more in the nanocomposite [9]. These results support the unique, strong, and effective actions of the Rv nanoparticles in the treatment of colorectal cancer [13].

## 3. Conclusions

Solving the problem using the optimum in-situ gel nanocomposite film of repetitive dosing due to high metabolism and instability of Rv formed the aim of the study. The drug release experiment, storage time effects, and drug encapsulation efficiency confirmed the choice of the NA-Sp-Rv-1200 film. The drug encapsulation efficiency of NA-Sp-Rv-1200 was 97.87 ± 0.051%, which might were related to the kneading and formulation preparation method. Each component’s grade, amount, percentage, way of formulation, and final dosage form confirmed the SR behavior. The optimum formulation release was less in the acidic media than at a pH of 6.5, which was the opposite of the Rv free-drug release. The drug release results were supported and validated using the swelling study, particle size analysis, FTIR, powder XRD, and SEM. The storage time effect experiment suggested that the NA-Sp-Rv-1200 might have solved the Rv physiochemical stability problems. The IC_50_ value of NA-Sp-Rv-1200 was significantly lower compared to that of Free Rv.

Furthermore, the flow cytometry analysis confirmed the significant apoptotic activity of NA-Sp-Rv-1200. The levels of biomarkers showed that NA-Sp-Rv-1200 could cause significant enhancement of apoptosis and cytotoxicity in HCT-116 cells. These results suggest that a long-acting Rv nanocomposite in in-situ gels could be a promising agent for treating colorectal cancer. However, the study requires further animal studies to demonstrate the effectiveness of prepared nanocomposite formulation in colorectal cancer and preclinical and clinical studies to evaluate its efficacy in human-based studies on risk/benefit ratio. However, similar research has discussed using NA and Sp to form an in-situ gelling nanosuspension of 5-Flourouracil by Abdullah et al. (2021). The formed nanosuspension was faster in release than the present optimum NA-Sp-Rv-1200 film at the different pH profiles. In addition, the size of the encapsulated 5-Flourouracil in the film was smaller than the present NA-Sp-Rv-1200 film [13].

## 4. Materials and Methods

Rv was gifted by Jamjoom Pharmaceuticals Company, Jeddah, Saudi Arabia. Sp and NA were purchased from Sigma Aldrich, St. Louis, MO, USA. A human colorectal cancer cell line was purchased from ATCC (Manassas, VA, USA). HCT 116 cells were grown in Dulbecco’s Modified Eagle Medium (DMEM) (Gibco, London, UK), which was supplemented with 10% fetal bovine serum (FBS), penicillin, and streptomycin. The cell line was grown to 70% to 90% confluency at 37 °C in a humidified atmosphere consisting of 5% CO_2_. All the other chemicals and reagents were of analytical grade.

### 4.1. Rv-Sp Wet Granulation

Rv and Sp granules were prepared with water in 5:3 and 5:6 ratios using a ERWEKA FGS II Wet Granulator by wet granulation methods. A definite amount of water was added that was enough to form a paste between the dry Sp and Rv physical mixture. After that, the formed paste was pass through the FGS II Wet Granulator with a size classifier. Then, the milling using mortar and pestle was for the clumps in the remaining granules. After all, the granule bed was passed through a 250 µm sieve to have a narrow size distribution, affecting the film encapsulation process. Afterward, the room temperature drying was used to avoid the Rv degradation and Sp hardening after oven drying [11,15]. The fume-hood was closed, and the air suctioning was accelerating the water evaporation for 12 h. The weight of the granules was measured after this optimized procedure and time to have a final dry granules weight equivalent to the added Sp and Rv weights ± 0.02 g. The solid dispersion containing 0.5 g of Rv was reconstituted with 15 mL of distilled water for the drug release experiment, represented in Table 1 as Sp 0.3-Rv and Sp 0.6-Rv.

### 4.2. Nano-Drug Composites in In-Situ Gelling Film Preparation

An NA solution of 10% was prepared using 350,000 to 400,000 g/mole (Sigma-Aldrich, Burlington, MA, USA). To be homogenized with the Rv:Sp (5:3 and 5:6) granules using a stirrer machine (ERWEKA PRS Planetary Stirrer, Germany), different volumes of the NA were optimized to achieve the most optimum SR profile and higher release in the intestinal-colonic simulated media, as listed in Table 1. These optimizations are for wound healing, and anti-inflammatory uses to manage inflammatory bowel diseases ulcerative colitis. In addition, these optimizations are directed for colorectal or systemic cancer management [8]. Thus, the Sp 0.3-Rv and Sp 0.6-Rv wet granules were homogenized with 0.43 g/g and 0.52 g/g, respectively (the optimum amounts), in the NA solution. After homogenization, the suspension was transferred and flattened on a glass plate. The process comprised a specific volume of the NA suspension containing the Sp-Rv granules to be poured on a circular glass plate with 6 ± 0.01 cm diameter using a fluid surface balance to ensure the uniform suspension distribution. The flattening procedure was by moving the plate to have 1 mm ± 0.03 mm thickness of the film. The films were left for over-night drying at room temperature. The room temperature drying was used to avoid the Rv degradation and Sp hardening after oven drying [12,15]. The fume-hood was closed, and the air suctioning was accelerating the water evaporation for 12 h. The weight of the granules was measured after the optimization for the procedure and time to have a final dry granules weight equivalent to the added Sp and Rv weights ± 0.02 g. These films were intended to be given orally as a dry film to be given with 15 mL of water, coded as NA-Sp-Rv-600 and NA-Sp-Rv-1200 [13,14,16].

### 4.3. In Vitro Drug Release Analysis and Drug Encapsulation Assay

**Figure 12:**
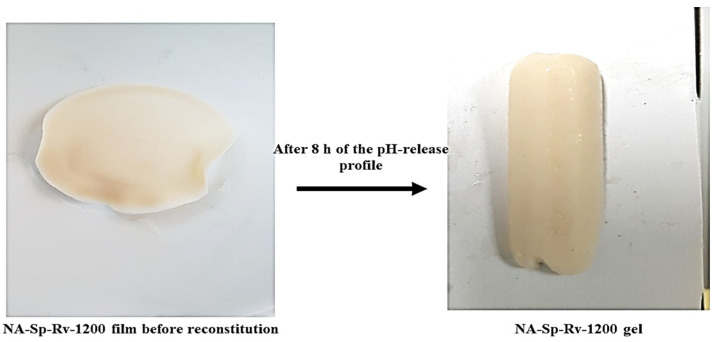
Illustration of long-acting resveratrol nanocomposite in-situ gelling film after 8 h of pH profile study.

The formulations in Table 1 containing 0.5 g of the Rv were placed into dialysis bags (14,000 Daltons cut off, Sigma-Aldrich, USA). The dry weights of the formulations were reconstituted with distilled water before enclosing them inside the bag. After reconstitution in water, the film will not gel with 15 mL/tablespoon of water. In addition, the in-situ gelling ability of the film started when it was in contact of 0.1N HCl [13,14]. This was simulated in the drug release experiment when the film was enclosed with a specific volume of distilled water in a dialysis bag. The optimum NA-Sp-Rv-1200 formula and gel formed were shown in Figure 12.

Moreover, the release media volume was 300 mL at 37 ± 0.5 °C in a shaking water bath at 75 rpm. The drug-release experiment was designed to simulate gastrointestinal pH and transit time after oral ingestion. As a result, the dialysis membranes were exposed to simulated gastric media (0.1 N HCl, pH 1.2), proximal intestine simulated media (phosphate buffer, pH 6.5), simulated media of the distal area of the small intestine (phosphate buffer, pH 6.8), and phosphate-buffer media of pH 7.4 of descending colon. The transit time for each simulated media was 2 h [13,14,21,22]. Total release media replacement was done after 1 h of dissolution to simulate sink conditions. The drug release was measured by ultraviolet (UV) absorbance at 306 nm [12]. The encapsulation efficiencies of formulations were measured by dissolving the formulation in ethanol and centrifuging it for 30 min at 1500 rpm after which an accurate volume of the supernatant transparent layer was measured by UV absorbance at 306 nm for Rv content using the equation below [13,26]. This experiment was conducted to optimize the formulation depending on sustained release behavior and drug encapsulation efficiency. The selected optimum formula was subjected to the kinetic fitting for the release, storage time effect, gel swelling studies, nanoparticles, and physiochemical characterizations.

$$Encapsulation\;efficiency=\frac{Total\;drug\;amount-Supernatant\;drug\;amount}{Total\;drug\;amount\;\left(mg\right)}\times 100\%$$

### 4.4. Storage Time Effect on the Nanocomposites of In-Situ Gelling Film

The most optimum formula (NA-Sp-Rv-1200) was stored at room temperature in an amber glass bottle containing a desiccant bag to decrease the film’s water uptake during storage. The drug release experiments for the formula were conducted after 2-, 4- and 8-weeks of storage [16].

### 4.5. Swelling Study of Nanocomposites of In-Situ Gelling Films–Rv Nanoparticles

The method relied on the film and gel weights that was presented in Figure 12 based on the reference [24]. Hence, a dry formulation film was weighed and then immersed in 300 mL of swelling media inside the dialysis bag, resembling the pH profile of the drug release experiment. After 2 h of exposure to each simulated medium, the film’s gel was removed and weighed. The swelling ratio was taken as the mean value of triplicate measurements. The swelling ratio was calculated using the following equation:

Q=(Ms−Md)/Md

Hence, *Q* is the swelling ratio, *Ms* is the gel mass after immersing inside the media, and *Md* is the dry film mass [24].

### 4.6. Nanocomposites of In-Situ Gelling Films-Rv Nanoparticle and Sp-Rv Granules Characterizations

#### 4.6.1. Particle Size Distribution and Zeta-Potential

The particle size and poly-dispersity index (PDI) of NA-Sp-Rv-1200 nanocomposite films were assessed using a Zeta-sizer (Nano ZSP, Malvern Instrument, Worcestershire, UK). The film was reconstituted in distilled water (Refractive index is 1.33) before measurements. The zeta-potential evaluation was done with disposable measurement cells (DTS 1070, Malvern). All measurements were obtained in triplicate.

#### 4.6.2. Chemical and Physical Interactions Elucidation

Solid sample characterization for interactions was performed using the FT-IR spectrophotometer (Thermo-Scientific, Nicolet-iS10, Waltham, MA, USA). The study samples were Rv, Sp, NA, Sp 0.6-Rv wet granules, films of NA-Sp-Rv-1200, and its physical mixtures (PM-NA 1200), Sp-NA films, and Rv-NA films. In addition, dry samples were subjected to compression using the instrument pin. The scan for the samples was set at a laser frequency of 15,798.7 cm^−1^ of medium resolution.

#### 4.6.3. X-ray Diffraction of the Wet Granules and In-Situ Gelling Films

The study samples were Rv, Sp, NA, Sp 0.6-Rv wet granules, NA-Sp-Rv-1200, PM-NA 1200, Sp-NA films, and Rv-NA films. They were characterized by the degree of crystallization using a high-resolution XRD (Maxima XRD-7000X, Shimadzu, Kyoto City, Japan). The XRD scanning speed was 5–80°/min.

#### 4.6.4. Dispersion, Morphology, and Distribution

SEM characterization of Rv, Sp, NA, Sp 0.6-Rv wet granules, and films with NA-Sp-Rv-1200 and PM-NA 1200 were performed using a microscope (FEI Inspect F50, FEI, Tokyo, Japan) and coater (Emitech K550X, Quorum Technology Ltd., Laughton, UK). For coating and metallization, they were coated under vacuum with silver and platinum on a metal stub. The potential used was 5.0 Kv, and the magnification power was 3000×.

### 4.7. In Vitro Cell-Line Study on HCT-116 Cancer Cells

#### 4.7.1. Cell-Viability Analysis Using MTT Assay

HCT-116 cells were seeded in 96-well plates at a density of 5 × 10^3^ cells/well and treated for 24 h with 0.40, 1.60, 6.30, 25, and 100 µg/mL Rv as free active material (positive control), NA-Sp-Rv-1200, and blank films compared to the untreated cells group (negative control). The cells were then treated with 10 µL of tetrazolium (MTT) solution and incubated at 37 °C for 4 h. After washing with phosphate-buffered saline (PBS), the precipitates were dissolved in 150 µL dimethyl sulfoxide (DMSO) for 10 min, and absorbance measurements were obtained at 563 nm. The relative cell viability with vehicle-treated/control groups for each concentration was determined by comparing the absorbance of each precipitate at 563 nm [25].

#### 4.7.2. Apoptosis Analysis Using Annexin V-Propidium Iodide Assay

The annexin-V staining technique was used to evaluate the apoptosis induced by Rv, NA-Sp-Rv-1200 film, blank, and untreated control cells. In 6-well plates, 1 × 10^5^ cells/well were seeded and incubated with IC_50_ concentrations of the drug and formulation for 24 h (37 °C) before being collected and centrifuged at 200× *g* for 5 min. They were washed twice after harvesting the cells and resuspended in PBS at room temperature. The mixture was incubated for 5 min at 25 °C with 10 µL annexin V-fluorescein isothiocyanate (FITC) and propidium iodide (PI) solution (5 µL). A flow cytometer was used for the study (FACS Calibur, BD Bioscience, San Jose, CA, USA) [25].

#### 4.7.3. Cell-Cycle Analysis

HCT-116 cells (1 × 10^5^ cells/well) were seeded and incubated for 24 h with Rv, NA-Sp-Rv-1200 film, blank, and untreated control group to determine cell cycle distribution. After 24 h, the medium was collected and washed twice with PBS, trypsin was applied, and collected the cells pellet. The pellet was rinsed with PBS, fixed in 70% ethanol, and stored at −20 °C overnight. According to the manufacturer’s instructions, the cells were rinsed with PBS to extract the alcohol before being stained with PI and treated with RNAase and then analyzed using flow cytometry [27,28].

#### 4.7.4. Gene Expression Analysis Using RT-PCR

RT-PCR was used to examine the expression levels of BAX in addition to B-cell lymphoma type 2 protein (*BCL-2*), tumor necrosis factor-alpha (*TNF-α*), and interleukins 1 beta (*IL-1β*) and *IL*-6 in the Rv, NA-Sp-Rv-1200 film, blank, and untreated control cells groups. The study used the Qiagen RNA extraction/BioRad SYBER green PCR package. For reading, a Rotor gene RT-PCR device was used. Rotor-Gene 1.7.87 program was installed on the machine [27,28]. Table 4 displays the primer sequences used in the analysis.

#### 4.7.5. Protein Concentration Assessment Using ELISA

Caspase-3 and Caspase-9 and P53 protein expression levels were measured in the Rv, NA-Sp-Rv-1200 film, blank, and untreated control cells groups using an ELISA. This assay was done with monoclonal antibodies against the corresponding protein (Ab-1), and the assays were performed according to the manufacturer’s instructions.

### 4.8. Statistical Analysis

The experiments were done in triplicate, and the results are presented as mean ± standard deviation. The statistical significance was determined using one-way analysis of variance (ANOVA) followed by the Tukey multiple comparison test with *p* < 0.05 considered to be significant.

## Figures and Tables

**Table 1:** Composition of formulations of Rv or Rv-Sp solid dispersions (Rv dose is 0.5 g) relative to product weight (g).

Code	NA-Level (g/g)	Sp-Level (g/g)	Film (F) or Physical Mixture (PM)	Rv Solid Dispersion (SD) or Free	Product Weight (g) Containing 0.5 g-Rv *
NA-Sp-Rv-600	0.43	0.21	F	SD	1.35 ± 0.015
NA-Sp-Rv-1200	0.52	0.26	F	SD	2.35 ± 0.013
PM-NA 1200	0.52	0.26	PM	Free	2.31 ± 0.011
Sp 0.3-Rv	-	0.38	-	SD	0.83 ± 0.031
Sp 0.6-Rv	-	0.55	-	SD	1.11 ± 0.023
Rv-NA	0.70	-	F	Free	1.71 ± 0.015
Rv	-	-	-	Free	0.50 ± 0.00

* For *n* = 3 ± standard deviation.

**Table 2:** Kinetics fittings for dialysis bag method of NA-Sp-Rv-1200 film.

Korsmeyer-Peppas (0–2 h) Mt/M∞ = K* t^n^	n = 0.47 ± 0.04 K = 0.029 ± 0.02 R^2^ = 0.992 ± 0.04
Zero-order (2–8 h) y = ax + b	Slope (a) = 1.50 ± 0.02 Intercept = −1.61 ± 0.01 R^2^ = 0.997 ± 0.02

**Table 3:** Storage time effect on NA-Sp-Rv-1200 drug release.

Comparison of Percentage Release	NA-Sp-Rv-1200	NA-Sp-Rv-1200 (2 Weeks)	NA-Sp-Rv-1200 (4 Weeks)	NA-Sp-Rv-1200 (8 Weeks)
Gastric % Release *	4.77 ± 0.03	4.80 ± 0.061	4.81 ± 0.025	4.70 ± 0.023
Proximal Intestine % Release *	5.01 ± 0.03	5.10 ± 0.037	5.09 ± 0.023	5.12 ± 0.011
Distal Intestine % Release *	2.72 ± 0.021	2.76 ± 0.031	2.61 ± 0.051	2.63 ± 0.034
Colon % Release *	2.13 ± 0.031	2.20 ± 0.011	2.10 ± 0.031	2.03 ± 0.014
Gastric % Release *	4.77 ± 0.031	4.80 ± 0.061	4.81 ± 0.025	4.70 ± 0.023
Intestinal-Colonic % Release *	9.86 ± 0.044	10.06 ± 0.014	9.80 ± 0.034	9.78 ± 0.044

* For *n* = 3 ± standard deviation.

**Table 4:** Sequences of primers used in RT-PCR assay.

Gene	Sequence
BAX: F	5′-CTGCAGAGGATGATTGCCG-3′
BAX: R	5′-TGCCACTCGGAAAAAGACCT-3′
BCL2: F	5′-GACTTCGCCGAGATGTCCAG-3′
BCL2: R	5′-GAACTCAAAGAAGGCCACAATC-3′
TNF-α: F	5′-GCAACAAGACCACCACTTCG-3′
TNF-α: R	5′-CTCAAGTCCTGCAGCATTC-3′
IL-1β: F	5′-TGTACCTGTCCTGCGTGTT-3′
IL-1β: R	5′-CTCCCAGGAAGACGGGCATG-3′
IL-6: F	5′-CAACCTGAACCTTCCAAAGATG-3′
IL-6: R	5′-ACTCATCTGCACAGCTCTGG-3′
B-actin: F	5′-AGAGCTACGAGCTGCCTGAC-3′
B-actin: R	5′-AGCACTGTGTTGGCGTACAG-3′

## Data Availability

The data presented in this study are available in article.
